# Case Report: Is Surgical Treatment Beneficial for Intracranial Basal Ganglia Cunninghamellamycosis Following Haematopoietic Stem Cell Transplantation?

**DOI:** 10.3389/fped.2022.831363

**Published:** 2022-05-27

**Authors:** Bixin Xi, Aiguo Liu, Xin Zhao, Yicheng Zhang, Na Wang

**Affiliations:** ^1^Department of Paediatrics, Tongji Hospital, Tongji Medical College, Huazhong University of Science and Technology, Wuhan, China; ^2^Department of Hematology, Tongji Hospital, Tongji Medical College, Huazhong University of Science and Technology, Wuhan, China

**Keywords:** cunninghamellamycosis, intracranial basal ganglia, HSCT, surgical treatment, benefits

## Abstract

Cunninghamellamycosis is an unusual but often highly fatal mucormycosis caused by *Cunninghamella bertholletiae*, which belongs to the basal lineage order Mucorales. It is especially fatal when the central nervous system is involved. So far, there are few reported cases of surgical treatment for intracranial mucormycosis in children after allogeneic haematopoietic stem cell transplantation (HSCT). The surgical management of deep-seated basal ganglia fungal lesions remains controversial, and its clinical benefits are not yet well established. Herein, we present a rare case of disseminated mucormycosis caused by *C. bertholletiae* involving the lung and intracranial basal ganglia after homologous leucocytic antigen-matched sibling donor HSCT. The patient was successfully treated for intracranial cunninghamellamycosis with neuroendoscopic surgery and systemic wide-spectrum antifungal treatment and achieved pulmonary recovery without recurrent *C. bertholletiae* infection or neurologic sequelae. Over the follow-up period of 13 months, there were no adverse events associated with the intracranial surgical debridement, and the patient remained in good health.

## Introduction

Mucormycosis is a rare, emerging infection caused by fungi of the order Mucorales. The most common agents of mucormycosis in paediatric patients are *Rhizopus* spp. (39.7%), *Lichtheimia* spp. (17.5%, formerly of the genera *Absidia* and *Mycocladus*), and *Mucor* spp. (12.7%) (1, 2). Genera of other Mucorales, such as *Cunninghamella bertholletiae* (6.3%), are less frequently reported (1, 2), though *C. bertholletiae* infection can be associated with an increased mortality rate in immunocompromised children (2, 3).

The rate of morbidity of mucormycosis varies depending on the underlying conditions and sites of infection in patients (4–6). As medical science advances, the patient populations undergoing solid organ and haematopoietic stem-cell transplantations have expanded rapidly, with increasing incidence of mucormycosis (7, 8). In more recent years, immune dysfunction after transplantation evolved as an important risk factor for mucormycosis (8–10). The most common clinical manifestations of mucormycosis in children are rhino-orbito-cerebral, pulmonary, cutaneous, and gastrointestinal (11, 12). Disseminated mucormycosis, especially involving the central nervous system (CNS), is relatively rare but often associated with mortality rates over 80% (4).

Generally, improved outcomes are related to more prompt species identification and the application of multidisciplinary treatment approaches involving aggressive surgical debridement and antifungal therapy (8, 13). Intracranial basal ganglia mucormycosis is a devastating neurologic disease with high mortality (4), and its management is still controversial. Despite active antifungal treatment, the expected clinical outcome remains poor, especially for large-volume lesions. Compared with conservative management, surgery may be more beneficial in paediatric cases for deep-seated intracerebral mucormycosis. Here, we report a successfully treated case of a 17-year-old boy who developed disseminated mucormycosis caused by *C. bertholletiae* involving the lung and basal ganglia following allogeneic haematopoietic stem cell transplantation (allo-HSCT). This study shows that neuroendoscopic surgery can safely and effectively remove deep-seated lesions and is a promising approach for intracranial mucormycosis. Additionally, during the 13-month follow-up period post-operation, the patient recovered well without recurrent *C. bertholletiae* infection or neurologic sequelae.

## Case Description

A 17-year-old boy with acute myeloid leukaemia (AML) received an allo-HSCT from a human leukocyte antigen-matched sibling donor in May 2020. The conditioning regimen consisted of cytarabine, busulfan, cyclophosphamide, and porcine antilymphocyte globulin (p-ALG), with graft-versus-host disease (GVHD) prophylaxis of cyclosporine A (CsA), mycophenolate mofetil (MMF), and short-course methotrexate (MTX). Oral voriconazole was administered as prophylaxis for possible fungal infection, maintaining the trough level between 2 and 5 mg/L after transplantation. Neutrophil and platelet engraftment was achieved on day 17 (day + 17) and day + 19 after allo-HSCT, respectively, and the patient was safely discharged on day + 20. The initial post-transplantation course was uncomplicated, and stable donor chimerism was achieved from day + 43.

On day + 52, the patient was hospitalised for nausea, vomiting, abdominal pain, and severe watery diarrhoea (35 ml/kg/24 h) without fever or liver dysfunction. Paediatric colonoscopy with multiple biopsies confirmed the diagnosis of overall grade IV acute GVHD in the gastrointestinal tract. Methylprednisolone (2 mg/kg/day), tacrolimus (1 mg/day), and basiliximab (20 mg/dose/week) were promptly administered intravenously to prevent worsening of GVHD, in addition to parenteral nutrition and anti-diarrhoeal agents. Clinical conditions gradually improved, and a second endoscopy showed a smooth colonic mucosa without any residual signs of GVHD. On day + 114, the patient was discharged from the hospital.

**FIGURE 1:**
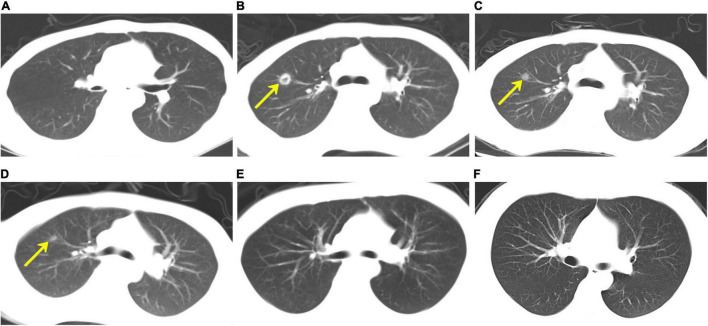
Computed tomography (CT) scan of the chest. **(A)** As part of his evaluation after haematopoietic stem cell transplantation, chest CT was unremarkable on day + 92. **(B)** On day + 123, the patient developed a fever and anorexia, and chest CT revealed a new right upper lobe defined nodule (17 × 15 mm) surrounded by a ring of consolidation. Chest CT scans, which were performed on days + 152 **(C)**, + 251 **(D)**, and + 519 **(E)**, respectively, demonstrated a gradual reduction of nodule size. **(F)** On day + 540, the chest CT revealed that the original right upper lobe nodule disappeared completely.

**FIGURE 2:**
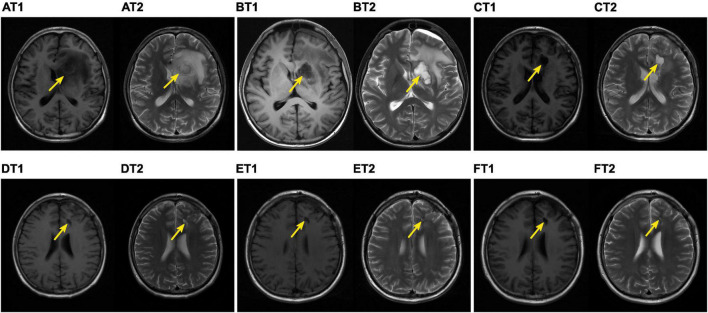
Brain magnetic resonance imaging (MRI) findings. T1, magnetic resonance T1-weighted imaging. T2, magnetic resonance T2-weighted imaging. On day + 138 after haematopoietic stem cell transplantation (HSCT), the patient experienced persistent headache and violent mood swings. The follow-up MRI-T1 **(A_*T*1_)** and MRI-T2 **(A_*T*2_)** identified signal abnormalities (see yellow arrows) suggestive of left-side basal ganglia abscesses (28 × 18 mm) and signs of perifocal oedema (pre-operation). On day + 141 after HSCT, intracranial debridement was performed on the child. On day + 164 after HSCT, MRI-T1 **(B_*T*1_)** and MRI-T2 **(B_*T*2_)** showed that the basal ganglia mucormycosis was completely removed post-operation. On day + 183 after HSCT, MRI-T1 **(C_*T*1_)** and MRI-T2 **(C_*T*2_)** were performed post-operation. On day + 216 after HSCT, MRI-T1 **(D_*T*1_)** and MRI-T2 **(D_*T*2_)** were performed post-operation. On day + 251 after HSCT, MRI-T1 **(E_*T*1_)** and MRI-T2 **(E_*T*2_)** were performed post-operation. On day + 339 after HSCT, MRI-T1 **(F_*T*1_)** and MRI-T2 **(F_*T*2_)** revealed that the surgical site appeared to be healing well with a gradual reduction in size.

On day + 123, the patient was readmitted for fever and anorexia without headache or diarrhoea. Computed tomography (CT) scan of the chest (Figure 1) revealed a new right upper lobe defined nodule (17 × 15 mm) surrounded by a ring of consolidation. Blood tests (Table 1) revealed a C-reactive protein level (CRP) of 208.9 mg/L [normal range (NR), ≤ 5 mg/L], with 7.19 × 10^9^/L white blood cells (NR, 3.5–9.5 × 10^9^/L) and 97 × 10^9^/L platelets (NR, 125–350 × 10^9^/L). Microbiological tests, including cultures and polymerase chain reaction (PCR) for pathogens, failed to identify any agents. Bone marrow evaluation revealed normal myeloid maturation without any signs of disease recurrence or acute GVHD and full-donor chimerism. He was treated empirically with intravenous voriconazole, acyclovir, imipenem, and linezolid. Three days later, the CRP level had decreased to 149 mg/L, but the patient’s condition did not improve, and the patient developed a cough. The patient’s blood sample was then sent to the Beijing Genomics Institute (BGI)-Wuhan for pathogen detection *via* next-generation sequencing (NGS). Two days later, NGS of the blood confirmed the presence of *C. bertholletiae* genetic material. Intravenous liposomal amphotericin B (LAmB, 1.14 mg/kg/day) and oral posaconazole delayed-release tablets (300 mg/dose, twice daily for 24 h loading dose, followed by 300 mg/dose/day maintenance) were promptly started on day + 132, along with a switch of antibiotic therapy from imipenem and linezolid to benzylpenicillin. Six days later, the patient’s temperature was normal, and the cough decreased noticeably, but he experienced persistent headache and violent mood swings. A follow-up brain magnetic resonance imaging (MRI; Figure 2) identified signal abnormalities suggestive of left-side basal ganglia abscesses (28 × 18 mm) and signs of perifocal oedema. No bacteria, viruses, *Mycobacterium tuberculosis*, or mycoplasma was detected *via* NGS in the cerebrospinal fluid (CSF), but *C. bertholletiae* was detected. CSF studies revealed negative minimal residual disease (MRD) with flow cytometry analysis. A diagnosis of overall disseminated mucormycosis caused by *C. bertholletiae* involving the lung and intracranial basal ganglia was made.

**TABLE 1:** Results of blood examinations.

Blood examinations	Day + 123	Day + 183	Day + 518	Normal range
White blood cells (10^9^/L)	7.19	5.85	4.28	3.5–9.5
Neutrophils (10^9^/L)	6.05	3.61	3.20	1.80–6.30
Haemoglobin (g/L)	112	75	128	115–150
Platelets (10^9^/L)	97	96	139	125–350
C-reactive protein (mg/L)	208.9	10.4	6.4	0–10
Alanine aminotransferase (U/L)	13	70	20	4–33
Aspartate aminotransferase (U/L)	22	34	25	4–32
Albumin (g/L)	32.3	40.8	48.8	35–52
Total bilirubin (μmol/L)	10.7	12.8	7.7	0–26
Total cholesterol (mmol/L)	5.78	2.32	3.38	< 5.18
Creatinine (μmol/L)	84	128	119	59–104
eGFR (ml/min/1.73 m^2^)	ND	69.9	76.4	> 90
PT (seconds)	14.0	14.5	12.7	11.5–14.5
APTT (seconds)	39.1	37.4	33.0	29–42
FIB (g/L)	5.41	2.34	3.21	2.0–4.0

*eGFR, estimated glomerular filtration rate; ND, not done; PT, prothrombin time; APTT, activated partial thromboplastin time; FIB, fibrinogen.*

A multidisciplinary team made the decision for surgical treatment for intracranial mucormycosis after assessing patient characteristics, the biology of *C. bertholletiae*, underlying disease, risks, and side effects. On day + 141, the patient was referred to the neurosurgery team for an intracranial debridement, along with the same antifungal therapy. NGS of the resected specimen was also positive for *C. bertholletiae* and confirmed the diagnosis of disseminated mucormycosis. After surgery, the patient’s clinical condition markedly improved, especially the fractious mood, and normal neurological function was restored without any other abnormalities. He recovered well post-operation without recurrent *C. bertholletiae* infection or neurologic sequelae. One month later, he was discharged with continued antifungal combination therapy outside the hospital. On day + 258, the intravenous LAmB and oral posaconazole delayed-release tablets were switched to maintenance monotherapy of posaconazole (300 mg/dose/day) for about 10 months. During the combination therapy, only mild adverse effects, including abdominal symptoms and anorexia, were observed, none of which necessitated the cessation of antifungal drugs. The patient showed good adherence and tolerance to posaconazole without any adverse events in the maintenance monotherapy. Throughout the treatment, he maintained a good clinical condition with no reoccurrence of symptoms.

At the 13-month follow-up visits, his neurologic examinations remained stable, and the surgical site appeared to be healing well with clean wound margins. The preoperative and postoperative lesion volume changes were evaluated using brain MRIs (Figure 2) and demonstrated a gradual reduction. Regular CT scans of his chest (Figure 1) revealed that the original right upper lobe nodule disappeared completely. Repeated NGS tests of the blood and CSF showed normal results. Currently, all laboratory test results (Table 1), except for slight serum creatinine increases, were determined to be within normal limits, and the patient was in good health.

## Discussion

Cunninghamellamycosis is a rare invasive fungal infection caused by *C. bertholletiae*, which is a subgroup of Mucorales (2, 14). Paediatric disseminated mucormycosis cases, especially *C. bertholletiae* infections after transplantation, are rare, but often reported with high fatality rates (4, 9). Here, we present a unique case of mucormycosis caused by *C. bertholletiae* involving the lung and intracranial basal ganglia following allo-HSCT, which was safely and effectively cured by neuroendoscopic surgery, in addition to the combination of LAmB and posaconazole. Furthermore, this is also the first study to precisely identify *C. bertholletiae via* NGS within 48 h, confirming its clear advantage over histopathological examination in diagnosis.

After aspergillosis and candidiasis, mucormycosis counts as the third most common invasive fungal infection in children with haematological malignancy, HSCT, and solid organ transplantation (9, 15). In recent decades, epidemiological studies have suggested an increasing incidence of mucormycosis in paediatric patients (12). Of the risk factors, immune dysfunction, an age of less than 1 year, selection pressure from primary prophylaxis with voriconazole, haematologic malignancy, iron overload, and parenteral hyperalimentation, particularly in children, have been reported to be the most common contributions to the growing morbidity of mucormycosis (12, 16). Jeong et al. reviewed 851 individual 18-year-old patient cases and found that corticosteroid use was the most common predisposing factor (33%), followed by neutropenia (20%), receiving cancer chemotherapy (18%), and the use of calcineurin inhibitors (16%) (17). In our case, the patient was immunocompromised after allo-HSCT and developed grade IV acute GVHD of the gastrointestinal tract. The neutropenia, colonic mucosa ulcers, and increased dose of the immunosuppressive regimen were assumed to be the most crucial predisposing factors for the disseminated *C. bertholletiae* infection. Therefore, early administration of anti-diarrhoeal agents, a strict diet, suitable doses of immunosuppressive drugs, and antifungal prophylaxis are crucial in order to rest the entire bowel and prevent gastrointestinal infections or GVHD.

A quick diagnosis of mucormycosis is challenging due to the often rapid invasion and destruction caused by this infection. Histopathology and culture of clinical specimens are the cornerstones of diagnosing mucormycosis in most cases, despite their time-consuming nature, which requires more than 1 week for results. It is difficult to distinguish Mucorales from *Aspergillus* spp. based on their morphological characteristics, and culture has poor sensitivity due to the fragility of Mucorales hyphae (12, 18, 19). Molecular examination improves the accuracy of species identification compared to morphological identification (12, 18). This case presents a novel diagnostic approach that used fresh blood and CSF preserved at 4°C, and biopsy tissue cryopreserved at –40°C for pathogen detections *via* NGS. Compared with histopathology and culture of clinical specimens, NGS testing requires less time, can be completed within 48 h, and has a better sensitivity for the identification of all potential pathogens (20), which are crucial to early diagnosis in patients with CNS disorders after allo-HSCT. Moreover, early diagnosis is very important for promptly initiating multidrug interventions, which are necessary for preventing progressive tissue invasion and minimising the demands of invasive surgery.

Currently, the optimal management of disseminated mucormycosis depends on a combined approach, including reversal of predisposing factors, early initiation of antifungal monotherapy or combination therapy, and the feasibility of complete surgical treatment (21). The 2019 Global guidelines for the diagnosis and management of mucormycosis from the European Confederation of Medical Mycology (ECMM) strongly support first-line antifungal treatment with LAmB across all patterns of organ involvement (8). LAmB possessed superior potency for combating most Mucorales strains but appeared to be less potent for *Cunninghamella*, and its renal toxicity can be a limiting factor (2, 15). Posaconazole is a novel member of the triazole class of antifungal agents effective against mucormycosis. The antifungal activity of LAmB in combination with posaconazole against the conidia of Mucorales was found to be significantly synergistic in an *in vitro* study (22). Results from some animal models and patient series have also shown the benefits of antifungal combinations in mucormycosis (12, 23–25). In our case, the application of early antifungal combination therapy between LAmB and posaconazole contributed to the normaliszation of temperature and decreased cough. However, the subsequent headache and violent mood swings confirmed that it was less effective against intracranial cunninghamellamycosis, probably due to the very low antifungal drug concentrations within the basal ganglia lesion. Moreover, the patient was unusually intolerant to the increased dose of LAmB (> 1.25 mg/kg/d), which caused severe renal toxicity.

Basal ganglia cunninghamellamycosis is extremely rare and difficult to access and presents a therapeutic challenge. On the one hand, surgical debridement of infected tissue or abscesses can remove areas with low antifungal drug penetration that might contain viable fungi, in addition to preventing contiguous intracranial spread and dissemination. On the other hand, invasive surgery for deep-seated lesions may injure the still functional cerebral tissue of the perifocal areas and lead to extensive damage. The European therapy guidelines strongly recommend that early surgical interventions—whenever possible—should be considered in patients with mucormycosis (8, 26). In a retrospective clinical study of 81 cases of CNS aspergillosis, patients who underwent neurosurgical interventions had significantly higher survival odds than those without neurosurgery (27, 28). Another study on rhinocerebral mucormycosis showed that early neurosurgical interventions are also associated with improved outcomes (25, 28, 29). Further studies on the surgical treatment of intracranial mucormycosis are urgently needed. Our case of basal ganglia mucormycosis was safely and effectively treated by neuroendoscopic surgery, which is unusual in paediatrics. The child recovered well post-operation without recurrent *C. bertholletiae* infection or neurologic sequelae. Follow-up brain MRI evaluations showed a healing surgical site, and neurologic examinations remained stable.

The total duration of antifungal therapy necessary to treat disseminated mucormycosis remains unknown. In general, months to years of therapy are empirically provided to immunocompromised patients. The European guideline group strongly supports treatment until permanent resolution of signs and symptoms of infection and complete response on imaging (8, 26), which might be difficult to determine due to patient characteristics and the extent of disease. In our case, the antifungal treatment will be continued for several months, then discontinued until there is permanent reversal of immunosuppression. We recommend a structured follow-up for the patient, consisting of neurologic examinations and brain MRI evaluations in the neurosurgery outpatient clinic, as well as visits with the infectious disease specialist and haematologist once every 6 months for 2 years. Furthermore, the child and his parents were satisfied with the treatment and would like to undergo follow-up visits regularly. The patient is currently stable.

## Conclusion

Disseminated mucormycosis in children, especially to the CNS, caused by *C. bertholletiae* is extremely rare, but typically progressive and frequently fatal. Surgical debridement, when needed and possible, should be considered as an urgent priority to prevent contiguous intracranial spread and dissemination, because it is likely to offer the best outcome and chance of survival. In addition, optimal antifungal therapies with good efficacy and acceptable toxicity are still an urgent unmet need, and also require further investigation regarding their clinical utility.

## Data Availability Statement

The original contributions presented in the study are included in the article/supplementary material, further inquiries can be directed to the corresponding author.

## Ethics Statement

This study was approved by the Ethics Committee of Tongji Hospital, Tongji Medical College, Huazhong University of Science and Technology. The study was performed in accordance with the Declaration of Helsinki, and written informed consent for the study and the publication of this report was obtained from the patient and his parents.

## Author Contributions

BX involved in patient management, follow-up study, data collection, data analysis, and manuscript writing and revising. AL and XZ performed data analysis and manuscript review. YZ and NW contributed to patient management, follow-up study, manuscript review, and data interpretation. All authors approved the final manuscript as submitted and agreed to be accountable for all aspects of the work.

## Conflict of Interest

The authors declare that the research was conducted in the absence of any commercial or financial relationships that could be construed as a potential conflict of interest.

## Publisher’s Note

All claims expressed in this article are solely those of the authors and do not necessarily represent those of their affiliated organizations, or those of the publisher, the editors and the reviewers. Any product that may be evaluated in this article, or claim that may be made by its manufacturer, is not guaranteed or endorsed by the publisher.
